# Targeting Neuroinflammation in Neovascular Retinal Diseases

**DOI:** 10.3389/fphar.2020.00234

**Published:** 2020-03-10

**Authors:** Tianxi Wang, Demetrios I. Tsirukis, Ye Sun

**Affiliations:** Department of Ophthalmology, Boston Children’s Hospital, Harvard Medical School, Boston, MA, United States

**Keywords:** neuroinflammation, neovascularization, retina, retinal disease, pharmacology

## Abstract

Retinal blood vessels provide the necessary energy, nutrients and oxygen in order to support visual function and remove harmful particles from blood, thus acting to protect neuronal cells. The homeostasis of the retinal vessels is important for the maintenance of retinal visual function. Neovascularization is the most common cause of blindness in patients with retinopathy. Previous studies have shown that inflammatory mediators are known key regulators in retinopathy, but their causal link has been elusive. Although inflammation is often thought to arise from inflammatory cells like macrophages, neutrophils, and resident microglia, retinal neurons have also been reported to contribute to inflammation, through inflammatory signals, which mediate blood vessel growth. Therefore, it is important to explore the detailed mechanisms of neuroinflammation’s effects on retinal neovascularization. This review looks to summarize current research on the relationship between retinal angiogenesis and neuroinflammation in retinopathy, as well as the potential effects of neuroinflammation on retinal neovascularization in different animal models.

## Retinal Neovascular Diseases and Neuroinflammation

The retina is part of the central nervous system and retinal blood vessels are functionally analogous to the cerebral blood vessels (Campbell and Humphries, 2012). The blood-retinal barrier (BRB) is formed by glial cells, pericytes and endothelial cells (Cunha-Vaz et al., 2011). Retinal blood vessels provide abundant energy and oxygen to neuronal and glial cells, while neuronal and glial cells provide growth factors for retinal blood vessels. In order to facilitate this, there is frequent and effective communication between neurons and vessels in the retina. Additionally, the BRB plays an important role in maintaining the function of the retina. An injury to the BRB causes neuroinflammation, which can result in BRB breakdown and neovascularization (NV). There is abundant evidence indicating that retinal NV is often accompanied by neuroinflammation (Connor et al., 2007; Sun et al., 2015, 2017), but how exactly neuroinflammation regulates retinal NV remains largely unknown.

Neuroinflammation causes neuronal damage, leading to the development and progression of a variety of neurodegenerative diseases such as Alzheimer’s disease, Parkinson’s disease, and retinal degeneration (Xing et al., 2016), as well as retinal neovascular diseases (Tonade et al., 2016; Sun et al., 2017). This damage triggers a rapid transformation of the retinal microglia into an activated state, switching its function from patrol to shield of the injured site (Chang et al., 2009). Activated microglia continue to secrete inflammatory mediators that act on other cells to induce and amplify uncontrolled inflammatory responses. Retinal neurons, such as photoreceptors, have been recently reported to signal for blood vessel growth through inflammatory signals (Tonade et al., 2016; Sun et al., 2017). Pro-inflammatory cytokines and chemokines can cause neuronal apoptosis or death (Leung et al., 2016).

Neovascularization is the proliferation of new micro blood vessels in the retina. In clinic, these are called intraretinal microvascular abnormalities, as well as retinal NV when these new blood vessels grow to the surface of the retina (Campochiaro, 2013). The biggest difference between normal retinal vessels and new blood vessels is that the new blood vessels lack tight junction proteins, which means that the plasma in the NV leaks into the surrounding tissue, such as the vitreous, and causes the degeneration of the vitreous, resulting in vitreous hemorrhage. Furthermore, the subsequent pull on the retina by degraded vitreous may result in retinal detachment, which involves the macula and results in severe vision loss (Campochiaro, 2013). NV occurs in many ocular diseases, such as retinopathy of prematurity (ROP), age-related macular degeneration (AMD), and diabetic retinopathy (DR). However, the causes of retinal NV may be different among the different types of retinopathy.

Choroidal NV (CNV) is the major cause of vision loss in neovascular AMD. CNV is a process that involves the participation of vascular and extravascular components, such that CNV results in a complex tissue, which is composed of blood vessels, glial cells, myofibroblasts, retinal pigment epithelia, and inflammatory cells (Spaide, 2006). Immune dysregulation and inflammatory processes have been linked with CNV pathogenesis both clinically and experimentally (Ambati et al., 2013; Kumar et al., 2014; Chen et al., 2016). The release of a series of pro-angiogenic factors may be one of the causes of inflammatory cells triggering angiogenesis (Noel et al., 2007). Neutrophil or macrophage depletion was shown to reduce CNV formation (Espinosa-Heidmann et al., 2003; Zhou et al., 2005). Similarly, macrophage depletion was associated with decreased vascular endothelial growth factor (VEGF) production in laser-induced CNV (Espinosa-Heidmann et al., 2003). The recruitment of blood-derived macrophages appears to be more associated with CNV than resident microglia by bone marrow transplantation experiments (Caicedo et al., 2005). In addition, photoreceptors can control proliferative angiogenesis by modulating photoreceptor inflammatory signals (Sun et al., 2017).

Retinopathy of prematurity is a major cause of blindness in children (Lutty et al., 2006; Tasman et al., 2006; Blencowe et al., 2012). With advances in neonatal care, smaller and more premature infants who are at high risk for ROP are saved; therefore, increasing the overall incidence of ROP. Currently there is no preventative treatment for ROP. To find new ROP treatments in addition to earlier preventative therapies, understanding the molecular mechanisms of ROP development becomes crucial. Photoreceptors have been reported to play an important role in the ROP pathogenesis (Akula et al., 2007a, b). Oxygen-induced retinopathy (OIR) is a classical and effective model for studying NV in the ROP (Smith et al., 1994; Connor et al., 2009). In this model, relative hypoxia leads to an increase in the expression of VEGF in the retina neurons and glial cells (Sun et al., 2015). At the same time, hypoxia can also cause neuroinflammation by activating microglia (Deliyanti et al., 2017). The continually activated microglia promote inflammation and VEGF expression, and eventually exacerbate NV.

Diabetic retinopathy is a common and complex diabetic complication. Hyperglycemia and dyslipidemia are closely related to the development of DR (Fu et al., 2016, 2018). NV often occurs in the later stages of DR (Klaassen et al., 2013; Hu et al., 2017). There are numerous angiogenic molecules that take part in regulated new-vessel formation in DR, including VEGF (Tanaka et al., 1997). The microglia in retinas are activated in animal diabetic models (Rungger-Brandle et al., 2000; Kezic et al., 2013). Recent studies show that inflammatory changes in photoreceptors influence pathological angiogenesis in DR (Du et al., 2013; Kern and Berkowitz, 2015; Liu et al., 2016; Tonade et al., 2017). A dysregulation of communications among neurons including the photoreceptors, vascular cells, and glial cells plays a major role in the pathophysiology of proliferative DR (Antonetti et al., 2012), which is characterized by neovascularization, neuroinflammation and neurodegeneration.

## Targeting Neuroinflammation in Neovascular Retinal Diseases

Although NV is the leading cause of blindness in eye diseases, including DR, AMD, and ROP, the detail mechanisms of the pathogenesis of NV are still not well understood (Zhang and Ma, 2007). Currently, ablation surgery, angiogenesis inhibitors and growth factor antibody therapy are the primary methods for NV treatment. Angiogenesis inhibitors include ranibizumab, bevacizumab and aflibercept. Bevacizumab is also known as Macugen, Avastin or Lucentis. All of the inhibitors have a similar function – they mainly inhibit the formation of new retinal blood vessels by inactivating VEGF (Avery et al., 2014; Fu et al., 2016). However, it is important to note that ablation surgery may cause damage to the retina, and anti-VEGF treatments may inhibit the growth of normal vessels and neurons (Fu et al., 2019). It is crucial to find new ways to treat retinal NV.

**FIGURE 1:**
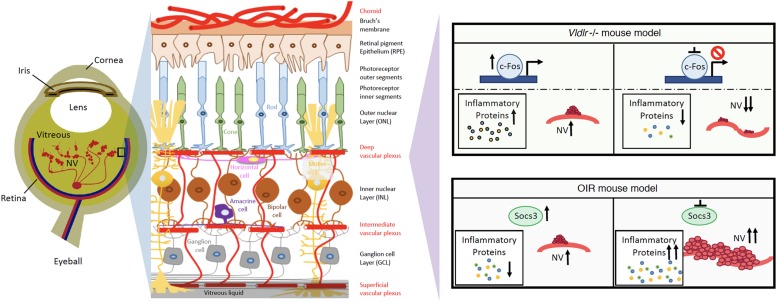
Schematics of neuroinflammation and neovascularization in retina angiogenesis mouse models. Targeting master inflammatory regulators, Socs3 or c-Fos, may provide new ways to reduce neuroinflammation and prevent the development of NV in retinopathies.

Because neuroinflammation is associated with retinal NV and promotes retinal NV (Fu et al., 2018), it may be possible to target neuroinflammation to treat retinal NV (Rivera et al., 2017). Recently, there have been attempts to suppress NV through controlling key regulators of inflammatory signals in the eye. Suppressor of cytokine signaling 3 (SOCS3) is an inducible negative feedback regulator of growth factor and inflammation signaling (Stahl et al., 2012), and plays a critical role in regulating inflammatory responses. SOCS3 is able to prevent pathological angiogenesis, and a conditional loss of SOCS3 in endothelial cells results in increased pathological NV (Stahl et al., 2012). The expression of SOCS3 is significantly increased in neuronal and glial cells in the OIR mouse model (Sun et al., 2015). A conditional knockout of SOCS3 in neuronal and glial cells exacerbates glial cell activation and neuroinflammation while promoting VEGFA expression and retinal NV (Sun et al., 2015). In addition, knocking out tumor necrosis factor alpha (TNFα), one of the inflammatory cytokines, appears to be protective in the OIR mouse model (Gardiner et al., 2005). Therefore, targeting the master regulator of cytokine signaling, SOCS3, may provide a new way to reduce neuroinflammation and suppress NV in the eye (Figure 1).

c-Fos is a proto-oncogene and is also important for many cellular functions. In human photoreceptor cells, c-Fos expression occurs throughout the development process (Yu et al., 1994). c-Fos regulates the expression of the rod-specific gene (He et al., 1998) and apoptosis in photoreceptor (Hafezi et al., 1997; Hobson et al., 2000; Poon et al., 2000). Additionally, c-Fos is strongly related to metabolic demands (Sun et al., 2017). In a retinal angiogenesis mouse model of very low-density lipid protein receptor (Vldlr) knockout mice, the expression levels of c-Fos and inflammatory cytokine (TNFα and Interleukin 6) are significantly increased in photoreceptors. Additionally, c-Fos controls retinal NV by modulating the neuroinflammation signals in this model (Sun et al., 2017). Therefore, targeting c-Fos may provide another way to reduce neuroinflammation in photoreceptors and prevent the development of NV in retinopathies (Figure 1).

In short, neuroinflammation is closely linked to angiogenesis, and improving the neuroinflammatory mechanism helps to inhibit retinal angiogenesis, while blocking inflammatory signals has also been reported to exacerbate retinal angiogenesis in the CNV model (Apte et al., 2006; Ambati et al., 2013). This happens because inflammation itself has two sides. In the early stages of neuroinflammation, activated glial cells work to clear damaged cells and protect the homeostasis of the retina. Persistent inflammation leads to a loss of control of glial cells, which in turn leads to an attack on their own cells through the secretion of inflammatory factors, exacerbating retinal angiogenesis. Therefore, inhibition of neuroinflammation to treat retinal angiogenesis is still controversial. To seek a better way to treat neuroinflammation, more investigation is needed to explore the detailed relationship between neuroinflammation and angiogenesis.

## Author Contributions

TW, DT, and YS contributed to the draft and edits of the manuscript. All authors approved the final manuscript.

## Conflict of Interest

The authors declare that the research was conducted in the absence of any commercial or financial relationships that could be construed as a potential conflict of interest.
